# Social mediatization of religion: islamic videos on YouTube

**DOI:** 10.1016/j.heliyon.2022.e09083

**Published:** 2022-03-10

**Authors:** Md. Sayeed Al-Zaman

**Affiliations:** a Department of Media and Technology Studies, University of Alberta, Edmonton, Alberta, T6G 2E6, Canada; b Department of Journalism and Media Studies, Jahangirnagar University, Savar, Dhaka, 1342, Bangladesh

**Keywords:** Mediatization, Social media, Islam, Waz, Online video, YouTube

## Abstract

Based on the conceptual framework of social mediatization of religion, this paper seeks to understand three pertinent phenomena: trends of online religious content, users' engagement with them, and correlations between interaction indicators. To answer the research inquiries, we quantitatively analyzed 73,120 Islamic videos uploaded on YouTube in 2011–2020. The result shows that Islamic videos on YouTube are growing continuously without any decline, from 6.04% in 2011 to 13.11% in 2019, more than two-fold in eight years. Also, with a strong positive correlation, comments and likes (*r* = .862; *p* < .01) are increasing at a remarkable rate than the other interaction variables. We observed that users who watch Islamic videos are more likely to like the videos than to dislike them. Users' engagement is not related to the length of Islamic videos in any significant way. These two results partly suggest users' positive and supportive attitudes toward online Islamic videos. Finally, this study recommends investigating the thematic temporal distribution of Islamic videos for an in-depth understanding of the users’ topical interest patterns.

## 1 Introduction: mediatization, social media, and religion

This paper employed the theory of social mediatization of religion as a conceptual framework to understand the patterns of religious communication in social media. Although *mediatization* is a widely used term, intellectuals from different schools of thought defined it differently. Their aggregate thoughts can be divided into two traditions: institutionalists and social constructivists (Deacon and Stanyer, 2014). While institutionalist tradition views mediatization as a process of adopting media by non-media actors, social constructivist tradition views it as the changing communication ecology of a society influenced by communication technologies. In general, mediatization can be perceived as the extension of media influence entrenched into almost every aspect of social life and that is connected to *culturalization* (Mazzoleni, 2008). The two main paradigms of media theories—media effects and media usage—have treated *media* as an independent entity and alien to society and culture (Hjarvard, 2011). Mediatization theory, thus, both transcends from and includes these two paradigms and redefines media as a social institution, responsible for the “interaction and transaction between actors and structures” (Hjarvard, 2011, p. 122). In the concept of mediatization, Mazzoleni (2008) addressed “media” as both a cultural technology and an organization. Although mediatization touches almost every sphere of social life, most of the prominent scholarly works on mediatization were more concerned with the mediatization of politics. In one of the earliest works, for example, Mazzoleni and Schulz (1999) investigated global politics and scholars' concerns regarding the growing intrusion of media into the realm of politics. Many scholars thought that media was weakening the functions and capacities of the traditional political institutions and elements. In contrast, the authors argued in this paper that mass media is nothing but reshaping political institutions, allowing political actors’ control on political processes and functions (Mazzoleni and Schulz, 1999, p. 247). In another notable study, Strömbäck (2008) discussed four phases of the mediatization of politics. The first phase indicates that media becomes the main source of information, whereas personal experience was the main source of information in the past. The second phase indicates that the media is independent of political power, whereas previously it was dependent on political institutions. The third phase indicates that media is now adopted by the political actors as well. The last phase discusses political and media logic: political actors were guided by political logic in the previous years, whereas they are now guided by media logic (Strömbäck, 2008, pp. 235–241).

On the one hand, these phases suggest that media has emerged as an autonomous and independent social institution, and on the other hand, media has integrated with the other social institutions (Hjarvard, 2008, 2011). Religion as a social institution has also been influenced by and become dependent on media recently, the theory of mediatization of religion suggests. Although religious mediatization is not a universal phenomenon, according to the first proposition of the theory, media has become an important and often primary source of religious information in many regions (Hjarvard, 2011). Second, media has become the source of religious experience as it now offers religious practices, beliefs, and symbols (Hjarvard, 2011; Lövheim, 2014). Third, “through their position in society media develop [*sic*] into social and cultural environments that take over many of the functions of institutionalized religions” (Lövheim, 2014, p. 551). As a result, a growing number of people are now relying on the media and adopting it for religious purposes. The internet-based media has now inaugurated a more convenient environment for the believers irrespective of their religions, making religion more inseparable and proximate to their everyday lives (Finnemann, 2011; Hjarvard, 2008). For example, in 2001, 25% of North Americans used the internet for religious purposes (Larsen, 2004), and the percentage reached 64% within the next three years (as cited in Al-Rawi, 2017). Though the statistics may justify it, the mediatization of religion through a new kind of media (e.g., internet-based media or social media) requires new explanations since the concept of mediatization was essentially built on mass media. Olsson and Eriksson (2016) labeled the new-age mediatization, which mainly relies on social media, as *social mediatization*. In the age of social mediatization, mass media logic should be replaced by four broad aspects of social media logic: programmability, popularity, connectivity, and datafication (Olsson and Eriksson, 2016; Van Dijck and Poell, 2013).

Though different social media platforms might influence different religions in different ways, this study is particularly interested in Islam. The Islamic use of online platforms has been addressed in previous studies (Al-Rawi, 2017), but empirical research on the trend of *social media*tization of Islam is insufficient. Emphasizing Muslims' social media practices and social mediatized Islam, Frissen et al. (2017) explored the dominant themes and symbols related to Islam on Instagram. They found that most of the Instagram posts contain quotes/text (38.1%) and photographs (33.8%). In terms of themes, most of the posts are directly related to Muslims' religious practices and beliefs (27.9%) (Frissen et al., 2017). Following a mixed method approach, Hazim and Musdholifah (2021) investigated the social mediatization of religion in Hungary. Although the local Islamic community's lack of integrity affects the Islamic mediatization, the study found, social media plays the most important role in this process. YouTube, followed by Facebook and Instagram, is the most popular source of Islamic information and experiences for the local Muslims. Weng (2019) studied Islamic preaching using social media in Indonesia and Malaysia, two Southeast Asian Muslim countries. This study offered three features of how social media is influencing Islam: It is allowing all Muslim users to equally express their religious feelings and ideas; Islamic scholars are equipping with adequate Islamic knowledge and proper communication skills; image, photo, video, color, and infographic in Facebook and Instagram are playing important role in preaching Islamic contents (Weng, 2019, pp. 92–93). However, this study barely emphasized YouTube as a popular social media platform harvested by Muslims. Al-Rawi (2017) studied extensively the use of YouTube in the Middle Eastern Islamic countries mainly from the aspects of Islamic extremism and various forms of cybercrimes (e.g., online flaming), but did not discuss enough about the essential communicative elements, such as growth, like, dislike, comment, and duration of Islamic videos on YouTube. Therefore, based on the previous literature, to provide some insights on the pertinent knowledge gaps, this study endeavored to answer the following questions:

*RQ1*: What are the changing patterns of online Islamic videos?
*RQ2*: What are the trends of users' interactions in online Islamic videos?
*RQ3*: How are the indicators of interactions related to each other?

The findings of this study suggest that Islamic content on YouTube is growing consistently with increased user engagement. The rest of this paper is divided into three sections. The next section describes the process of data collection and data analysis. The result section mentions the key findings of this study. The last section discusses the findings and their limitations and significance.

## 2 Materials and methods

This study endeavored to analyze online videos of Islamic preaching. For that reason, we chose the videos of *waz*, a type of Islamic preaching that becomes popular in contemporary Bangladesh with roots in the 19^th^ Century (for more, see Stille, 2021). We selected Bangladesh purposively (Bryman, 2012), mainly due to two reasons. Firstly, Islamic contents and sentimentalism are shifting from offline to online in Bangladesh thanks to the higher social media penetration and Islamic resurgence (Islam and Islam, 2018; StatCounter, 2021a), making the context worth exploring. Secondly, waz is an exclusive Islamic popular cultural element of Bangladesh that has a significant historical background and contemporary contribution to religious communication and practices. Moreover, waz sermons have become entrenched into a large number of Bangladeshi Muslims’ lives and exert considerable impacts. *Waz* is a type of Islamic oration containing “the glory of Islam and the greatness of its Prophet” (Hasan, 2012, p. 64), mainly delivered by the local Muslim scholars known as *Huzur*, *Mullah*, *Pir*, and *Maulana*. The public gathering in which a waz is delivered is called *waz mahfil*. Although waz mahfils are more commonplace in the rural areas of Bangladesh, it is now becoming popular in small towns and large cities as well. Besides preaching naïve Islamic sermons, waz mahfil often has links to the political Islamists and extremists who have communal intentions. Recently, the police found that Hefazat-e-Islam, an Islamic group, was controlling waz mahfils for political purposes (BDnews24.com, 2021). However, Hashmi (2000) addressed such waz mahfil as the product of illiterate and half-illiterate *Mullahs*, which might have negative social consequences. For example, waz mahfils preach intense misogynistic remarks against women and girls and promote socioreligious superstitions (Hashmi, 2000; Iftakhar, 2020). To reduce the extent of provocative statements, the government has taken initiatives to monitor waz mahfil*s* and their preachers (The Daily Star, 2021). Beyond the binary views, waz is rich with communicative ornaments. For example, the preachers aimed at influencing public emotions, and to achieve this goal they must use some unique rhetorical elements, including “long-cherished cultural forms and idioms,” and “poetry and songs that are well-known from Islamic education and from folk genres” (Stille, 2021, p. 297). Also, they use a particular form of chanting that distinguishes waz from other forms of communication. For the discussed reasons, waz has considerable political, cultural, social, and communicative significance.

The data for this study was collected from YouTube, a popular video-sharing social media platform (Aichner and Jacob, 2015). In Bangladesh, 5.94% of the total social media users use YouTube, which is the third most popular social media, preceded by Facebook (79.36%) and Twitter (10.54%) (StatCounter, 2021b). We selected YouTube as the source of data due to three reasons: Media scholars to date have not paid proper attention to YouTube, its relation to Islam, and the trend of YouTubed Islam; waz videos are usually longer that often exceed 2 h and they are convenient to upload on YouTube instead of any other platform; YouTube hosts a remarkable number of Islamic channels that is increasing rapidly (Al-Rawi, 2017; Frissen et al., 2017; Hazim and Musdholifah, 2021; Md Shahidul Haque, 2016; Weng, 2019). Using YouTube Data Tools (https://tools.digitalmethods.net/netvizz/youtube/), we extracted 88,470 videos using two keywords: waz (in both the English and the Bangla alphabet). Rieder (2015) developed this automated scraping tool to help the researchers who want to harvest YouTube data easily. Although a few previous studies used it for scraping YouTube data (e.g., Matamoros-Fernández, 2017), it is still an understudied API tool (Arthurs et al., 2018).

Our search criteria included three main components: a time range from 1 January 2011 to 31 December 2020, a 10-year span; region as Bangladesh; language as English and Bangla for two different search queries. Of the total extracted videos, we found 273 videos with missing data. Also, we excluded 16,350 duplicate and irrelevant videos through both automatic and manual processing. Our final sample included 73,120 videos and their metadata. These videos were published by 18,927 YouTube channels (Mean [M] = 3.86). In this study, we analyzed the six following metrics: number of videos, their views, comments, likes, dislikes, and durations (durations were measured in seconds). These are the basic analytics metrics of YouTube (Google, 2021). Prior studies also utilized these metrics in both descriptive and inferential statistical analysis (Alias et al., 2013; Bärtl, 2018). Important to note that YouTube waz videos are originated from waz mahfils that require a physical gathering. However, in 2020, such public gatherings were greatly hampered by the COVID-19 pandemic. Although our selected time span contains the year 2020, we less emphasized it in our discussion. To answer both RQ1 and RQ2, we devised a simple trend analysis of videos (Hess et al., 2001) with one-year intervals and produced a visualization using Tableau 2020.4 (Tableau Software, Seattle, WA, USA), a popular data visualization software. Previous studies with social media data also conducted similar trend analyses (Lau et al., 2012; Mehmood et al., 2014; Sakaki et al., 2011), making it an acceptable and standard method for exploring temporal patterns. To answer RQ3, we used IBM Statistical Package for the Social Sciences (SPSS) version 25 (IBM Co., Armonk, NY, USA) for performing an inferential statistical analysis, i.e., Pearson correlation coefficient analysis.

## 3 Results

### 3.1 Trend analysis

**Figure 1:**
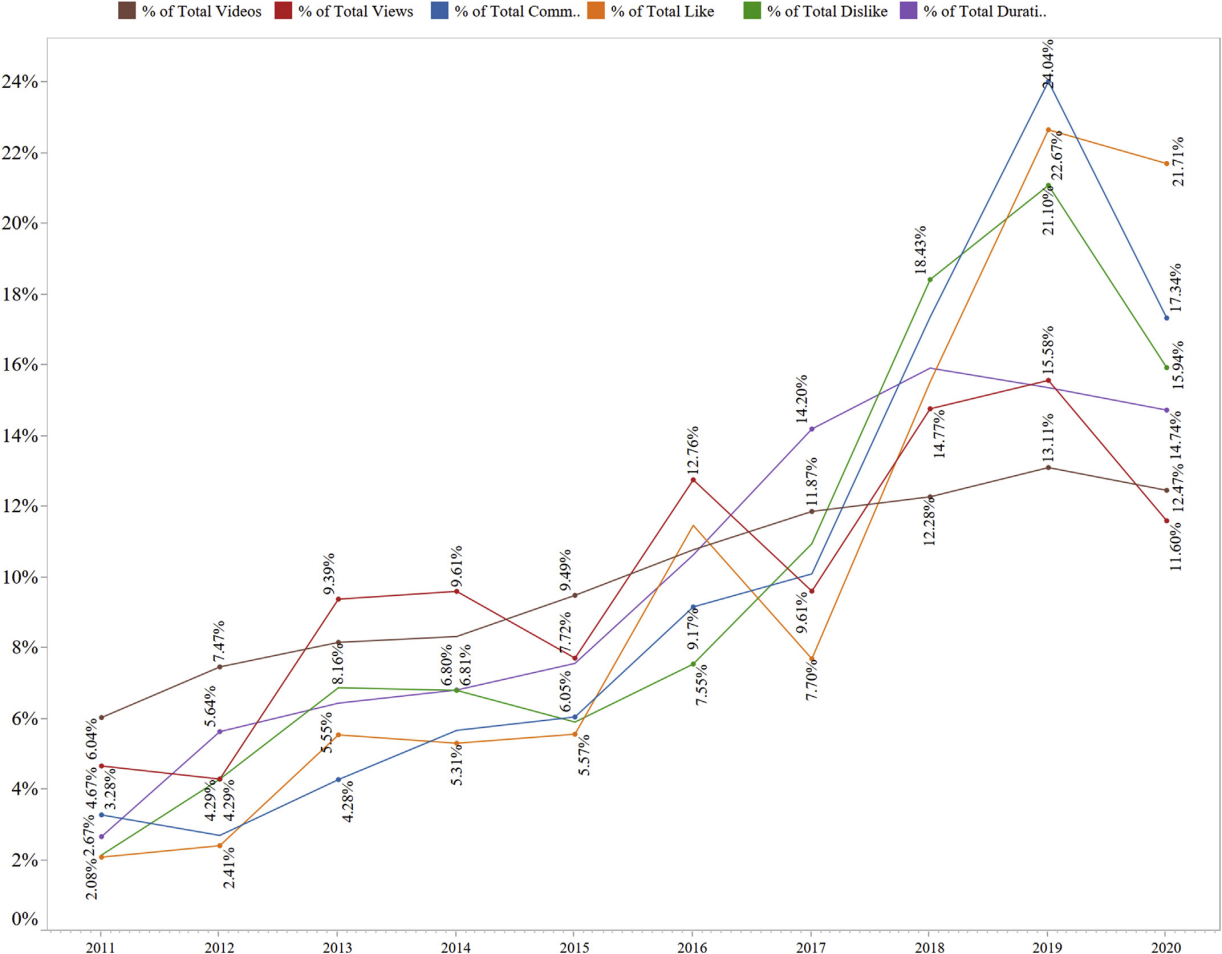
Trends of the variables during the selected study period.

The trend lines show that the growth of waz videos was increasing continuously during the period (Figure 1). From 6.04% in 2011, it reached 13.11% in 2019 following an uninterrupted growth. The growth was steadier until 2014, which accelerated afterward until 2019. In 2020, due to the COVID-19 pandemic, as mentioned earlier, public gatherings were prohibited by the government. This might be the reason for the reduced percentage (12.47%) of the waz videos in 2020. The aggregate percentage of durations mostly corresponded to the aggregate percentage of videos until 2016. Starting from 2.67% in 2011, video duration reached 7.57% in 2015. In the subsequent years until 2018 (15.92%), the percentage increased considerably before experiencing a minor drop in 2019 (15.37%).

Interestingly, although the percentage of videos and to some extent durations, was increasing continuously, the aggregate percentage of views of the videos fluctuated remarkably, following no specific patterns. For example, the views decreased from 4.67% in 2011 to 4.29% in 2012, increased in 2013 (9.39%), and again decreased in 2015 (7.72%). Similar oscillations continued in the rest of the years. We observed that users’ comments, an important indicator of online communication, were increasing continuously like the number of videos, except from the year 2012 (2.70%). In total, the percentage of comments reached 24.04% in 2019 from 3.28% in 2011, which was a remarkable growth.

Likes and dislikes are two other important indicators of online interaction. In general terms, the like button expresses appreciation, approval, likeness, and similar pertinent positive emotional reactions, whereas the dislike button expresses the opposite, i.e., disdain, dislike, disapproval, and other pertinent emotional reactions. The aggregate percentage of likes in the videos fluctuated frequently like views. From 2.08% in 2011, it reached 5.55% in 2013 before a slight decrease in 2014 (5.31%). The percentage increased and decreased in 2016 (11.47%) and 2017 (7.70%) before its highest surge in 2019 (22.67%). In contrast, the growth of dislikes was smoother than the trend line of likes. Starting from 2.14% in 2011, the percentage of dislikes reached 21.10% in 2019 with the only drop in 2015 (5.91%).

From 2011 to 2019, the aggregate percentage of videos increased by 7.07%, views increased by 10.91%, durations increased by 12.7%, comments increased by 20.76%, likes increased by 20.59%, and dislikes increased by 18.96%. Again, of the top three variables, likes decreased by 0.96% in 2020, whereas dislikes decreased by 5.16% and comments by 6.7%.

### 3.2 Correlation coefficient analysis

A Pearson correlation coefficient analysis between the five selected variables (i.e., duration, view, comment, like, and dislike) shows that view was most strongly and positively correlated with like (*r* = .862; *p* < .01), followed by dislike (*r* = .829; *p* < .01), and comment (*r* = .780; *p* < .01) (Table 1; Akoglu, 2018). Again, like was strongly and positively associated with comment (*r* = .779; *p* < .01), followed by dislike's moderate and positive correlation with comment (*r* = .640; *p* < .01). Like and dislike were also moderately and positively correlated (*r* = .603; *p* < .01). The duration of waz video was negatively correlated with both like (*r* = -.010; *p* < .05) and view (*r* = -.009; *p* < .01). However, their *r*-values indicate zero or negligible correlation between them. Similarly, duration is not correlated with dislike and comment.

**Table 1:** Correlations between the interaction variables.

Variables	Total (million)	M	SD	Duration	View	Like	Dislike	Comment
Duration	84.40	1154.29	1057.704	1	-.009∗	-.010∗∗	-.003	-.006
View	12042.12	164689.88	4270270.664	-.009∗	1	.862∗∗	.829∗∗	.780∗∗
Like	95.19	1301.84	21212.157	-.010∗∗	.862∗∗	1	.603∗∗	.779∗∗
Dislike	9.01	123.27	2102.342	-.003	.829∗∗	.603∗∗	1	.640∗∗
Comment	4.45	60.91	715.380	-.006	.780∗∗	.779∗∗	.640∗∗	1

∗ Correlation is significant at the 0.05 level (2-tailed).

∗∗ Correlation is significant at the 0.01 level (2-tailed).

## 4 Discussion and conclusion

This study aimed to answer three interrelated research questions regarding Islamic videos on YouTube. The results from the quantitative analysis suggest that Islamic videos on YouTube are growing continuously every consecutive year without any decline, and the recent surge is more conspicuous than the previous years. It supports the main tenet of the social mediatization theory of religion, that is, social media is impacting the prosumption (production and consumption; Beer and Burrows, 2010) of Islamic contents and reshaping the previous practices of Islam. Therefore, like Hazim and Musdholifah (2021) and Weng (2019), the present study also infers that YouTube can play an important role in mediatizing Islam digitally. One explanation of this growth of content production and consumption could be the increased religiosity of social media users. Users' diverse Islamic practices in social media platforms (e.g., sharing Quranic text, hadith, preaching) seem more visible in recent years, along with profuse production and consumption of Islamic contents (Al-Zaman, 2020). A recent survey shows that nine million people from Bangladesh joined various social media platforms between 2020 and 2021 (UNB, 2021). With them, Bangladesh has now 45 million social media users, which is 27.2% of the country's total population (UNB, 2021; We Are Social, 2021). Thus, it positioned at 8^th^ in the social media users' growth ranking with a growth rate of 25% (We Are Social, 2021). Therefore, from another point of view, it could also be possible that the growth of Islamic content is a result of the increasing number of social media users. Further studies are required to explicate these interrelationships empirically.

The growth of durations, comments, and dislikes are consistent with the growth of videos. On the other hand, the growth of views and likes increases with remarkable fluctuations. In terms of aggregate growth rate, comments and likes increase the most but their constancy may differ considerably. The likes in Islamic videos increase more than the dislikes. Again, the correlation analysis suggests, views and likes in most cases increase simultaneously. Dislike and comments also increase and decrease with view counts but are less likely than likes. It suggests that users who watch Islamic videos are more likely to like the videos than to dislike them. However, this fact must not be ignored that view counts in YouTube increase once a user accesses the video webpage, while likes, dislikes, and comments require users to log in to the website (Cheng et al., 2008). From another account, videos receiving more likes than dislikes from users are more likely to receive more comments. These findings are novel, suggesting likes and views could be two important indicators to understand users’ positive engagement with Islamic videos. The growth patterns and correlations of likes compared to dislikes implies that more users tend to appreciate and enjoy Islamic videos than to disapprove and deny them.

An interesting finding of this study infers that the duration of Islamic videos has an insignificant role that barely influences other interaction indicators. It further suggests that users’ engagement, both positive and negative, is less likely to depend on the duration of videos. Cheng et al. (2013) analyzing 15 categories of YouTube videos found that video duration and view counts are correlated but weakly. We found an insignificant or zero correlation of video duration with views and likes (Akoglu, 2018).

The results indicate users' apparently and increasingly positive attitudes toward Islamic YouTube videos. But what could be the underlying causes? Although it might not be within the scope of this study, we try to offer some insights that can guide future researchers in this regard. Since social media is not alien to real society, rather it has reciprocity with diverse social affairs, we must reconsider the contemporary socio-religious climate of Bangladesh to explain these issues. With the brand new “Islamic revivalism” (Islam and Islam, 2018), Bangladesh's existing public sphere has recently been experiencing a paradigm shift. Riaz (2013) and Rahman (2019) addressed it as the making of an *Islamic public sphere*. They included and emphasized the role of Islamic fiction, *halaqa* and *taleem* (Islamic gathering), madrassah, Islamic da'wa, Islamic parties, and social Islamization in their discussions. However, the social mediatization of Islamic practices, beliefs, and symbols with the help of emerging social media is absent in their studies, making them less effective to understand the contemporary religious communication climate and Islamic public sphere in Bangladesh. Therefore, we suggest more in-depth and empirical research on Islamic mediatization emphasizing social media's contribution in this process. In this regard, borrowing the idea from Hirschkind (2006), we presume that such online Islamic videos, an important element of contemporary Bangladeshi popular culture, could serve social media users as an instrument of ethical self-improvement and as a way of pious living.

To conclude, the results from this study would offer some insights to measure and understand how believers respond to online religious content. It also attempts to fill an important scholarly gap regarding online Islam in Bangladesh. With some novel findings, however, this study is limited within the quantitative domain, not explaining the underlying causalities of the identified trends properly. Also, cleaning such a big dataset precisely is difficult, and we suspect that some faulty data remained during the analysis, which could have minor impacts on the results. Apart from methodological constraints, this study only purposively focused on Bangladesh, not other Islamic countries such as Pakistan and Indonesia, leaving a gap for future research. Therefore, we recommend not overemphasizing these results and overgeneralize them for other Islamic countries. Considering the limitations, we further recommend a more in-depth theme analysis of the most popular Islamic videos, their thematic distributions throughout the time, and users’ engagement patterns with them.

## 5 Ethics and consent

No ethical issues.

## Declarations

### Author contribution statement

Md. Sayeed Al-Zaman: Conceived and designed the experiments; Performed the experiments; Analyzed and interpreted the data; Contributed reagents, materials, analysis tools or data; Wrote the paper.

### Funding statement

This research did not receive any specific grant from funding agencies in the public, commercial, or not-for-profit sectors.

### Data availability statement

Data will be made available on request.

### Declaration of interests statement

The authors declare no conflict of interest.

### Additional information

No additional information is available for this paper.
